# Assessment of Condyle-Sigmoid-Coronoid triangle, Gonial angle for age estimation and sex determination: A retrospective Orthopantomograph study

**DOI:** 10.1016/j.jobcr.2026.101411

**Published:** 2026-01-28

**Authors:** K. Smrithy Sivadas, Kumuda Rao, Vidya Ajila, Yashika Jain

**Affiliations:** Nitte (Deemed to be University), AB Shetty Memorial Institute of Dental Sciences, Department of Oral Medicine and Radiology, Mangalore, India

**Keywords:** Gonial angle, Condylar angle, Coronoidal angle, Sigmoidal angle, Forensic Odontology, Orthopantomograph

## Abstract

**Background:**

Due to its sexual dimorphism, the mandible—a vital component of the human skull—is a valuable tool for sex determination in anthropological and forensic studies. Radiographs like an Orthopantomogram are quite useful in this regard for precise age and sex estimation. An essential component of the craniofacial complex, the mandibular angles provide information about the symmetry and vertical characteristics. Variations in the Condylar, Coronoidal, Sigmoidal, and Gonial angles have been observed with age, sex, and even dental status, as verified by radiographic and anthropometric research.

**Aim:**

To evaluate Condylar angle, Sigmoidal angle, and Coronoidal angle by devising the Condyle-Sigmoid-Coronoid [CSC] Triangle along with the Gonial angle for sex determination and age estimation on a mandible devoid of teeth.

**Materials and methods:**

200 Orthopantomograph images were attained, categorised into Group I and Group II, which consisted of males and females with ages ranging from 10 to 80 years, respectively. The CSC Triangle was devised by joining the highest point on these landmarks.

**Results:**

The Condylar angle, Coronoidal angle, Sigmoidal angle composing the CSC triangle and the Gonial angle bilaterally were comparable between males and females with no statistically significant differences. The Condylar and coronoid angle had a higher score on the left side with the data demonstrating statistically significant results. The Sigmoidal angle and the Gonial angle were comparable between the right and left sides

**Conclusion:**

Based on the statistical analysis, the difference noted with respect to the measured parameters indicates that these angles can help in sex determination and age estimation.

## 1 Introduction

Determining age and sex by anthropometric measures utilising the mandibular features is a significant area of research in Forensic Anthropology. An individual's biological profile can be accurately and precisely ascertained from their skeletal remains using this method. The mandible, which undergoes distinct morphological changes throughout a person's life, is one of the primary skeletal elements used to determine an individual's age. Its value as a biometric is further enhanced by its inherent sexual dimorphism, which serves as the basis for sex estimation.^1^^,^^2^ The human mandible has undergone predictable evolution, with noticeable morphological changes at different stages of life. Like other bones in the body, the mandibular bone is impacted by hormonal fluctuations, age, and metabolic activity. Due to marked differences in the stages of mandibular development, growth rates, and longevity between the sexes, the mandible can distinguish between them. Furthermore, the size and shape of the mandible are influenced by the varied masticatory forces that men and women exert. Furthermore, it is believed that the occlusal condition and age of the individual affect the morphological alterations of the mandible, as longitudinal investigations have demonstrated that mandibular bone remodeling happens with age.^3^

The flat triangular plate that protrudes upward and slightly forward is called the coronoid process, whereas the condyle is a rounded projection that articulates with the temporal bone's glenoid fossa. The morphological appearance of the condyle can vary significantly based on age, sex, and other characteristics. Trauma, developmental variability, malocclusion, and other factors might potentially result in morphologic alterations.^4^ The deep notch that separates the coronoid and the Condylar process is known as the sigmoid notch. The form of these processes determines the notch's shape.^5^ The sigmoid notch, coronoid process, and condyle are all crucial anatomical features. The expression of mandibular growth in connection with age, sex, facial type, occlusal force, and functional load is provided by them. Given that it is the most resilient facial skeleton and maintains its shape better than other bones, the coronoid and condyloid appendages of the mandibular bone are thought to be appropriate for forensic investigation.^6^

Population-specific osteometric standards are necessary for accurate sex assessment due to varying skeletal features between populations.^7^ Panoramic radiographs (OPG) are the most commonly used extraoral radiographs, providing detailed views of hard tissue in the maxilla and mandible. This is the gold standard of care for dental screening, diagnosis, and treatment planning. It offers valuable information regarding the dentition and supporting bone.^8^ The common problems arising on a crime scene, from the collection of evidence up to producing a Dental Age Estimation report is that; numerous times the jaws are devoid of tooth or teeth sets required for age estimation. This may be due to missing, dissociated, decomposed and/or destroyed maxillofacial parts or further due to lack of awareness or knowledge of the collection of teeth as evidence for forensic examinations.

The present study was undertaken with an aim to evaluate various mandibular parameters for sex determination and age estimation by fabricating the Condyle-Sigmoid-Coronoid [CSC] Triangle in Orthopantomograph images, along with Gonial angles assessment. The objective of the study was to derive a formula for the age estimation using the radiographic parameters of the mandible devoid of teeth that could supplement the existing age estimation methods in the field of Forensic Odontology.

## 2 Materials and methods

### 2.1 Sample calculation

Based on Standard deviation of Condylar, Coronoidal angle in males 8.95 and in females 9.59; mean difference 5; effect size 0.5543; alpha error 5 %; power 90 % for 2-sided test; sample required is 69 per group i.e. a total of 138. This is calculated using n-Master's software version 2.

### 2.2 Study design

This retrospective study was carried out after obtaining the institutional ethical clearance (ETHICS/ABSMIDS/569/2025). 200 digital Orthopantomographs were acquired from the Romexis software database from January 2024 to January 2025 and consisted of 100 males and females scans with age ranging from 10 to 80 years. The scans were standardised at exposure parameters 66 kVp 9 mA and 16 s and modified based on the body type and age of the patient. Unclear or incomplete scan with artefacts and patients with history of Condylar fractures, severe developmental anomalies like micro-or macrognathia, TMJ ankylosis and any bony lesion as well as scans of patient with restricted mouth opening secondary to pathologies like premalignant conditions like Oral Potentially Malignant Disorders and Oral Malignancies were excluded from the study.

**Fig. 1:**
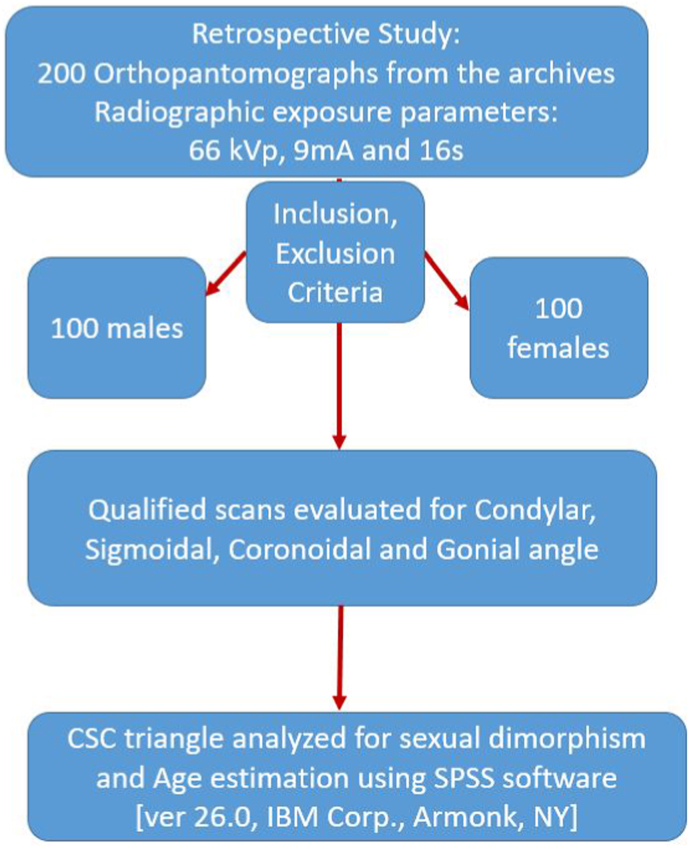
The flowchart depicting the methodology.

The radiologist who assessed the scans was blinded about the clinical diagnosis, age and sex of the patient. Measurements were done using the measurement tools of the Romexis imaging software. The measurements were made by a single radiologist and repeated after an interval of 2 weeks. The average of the measurements was considered as the final value thereby minimizing the Intra-Observer Bias. Qualified scans were evaluated for Condylar, Sigmoidal, Coronoidal angle for the purpose of evaluating the CSC triangle along with Gonial Angle. The flowchart depicting the methodology is given in Fig. 1.

**Fig. 2:**
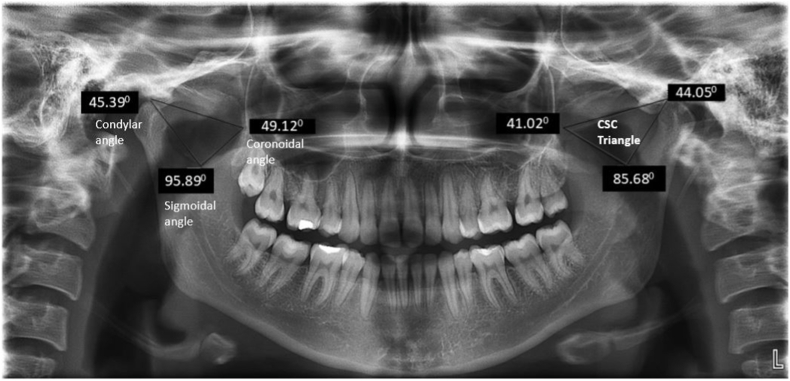
Measurements of angles in Condyle-Sigmoid-Coronoid [CSC] Triangle.

**Fig. 3:**
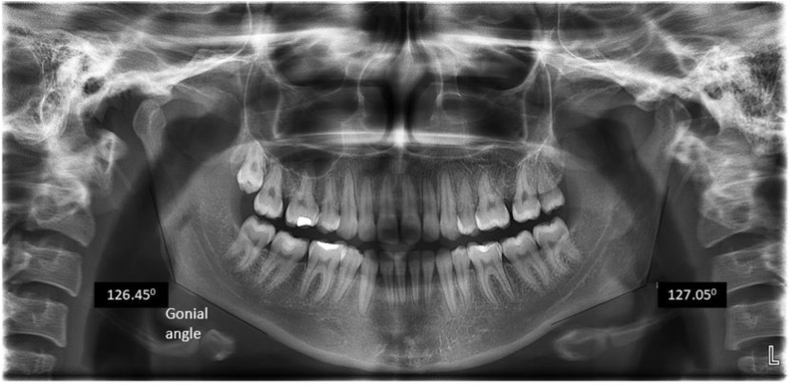
Measurement of Gonial angles bilaterally.

The parameters assessed were (Fig. 2, Fig. 3):

i) **Coronoidal angle**-measured by joining the highest point of the condyle to coronoid process to deepest point of the sigmoid notch
ii) **Condylar angle**-measured by joining the highest point of the coronoid process to condyle and to deepest point of the sigmoid notch
iii) **Sigmoidal angle**: Measured by joining the highest point to that of condyle to deepest point of the sigmoid notch to coronoid process
iv) **Gonial Angle-**measured by drawing a tangent to the lower border of the mandible and a tangent to the distal border of the ramus and condyle.

Coronoidal angle, Condylar angle, and Sigmoidal angle composed the 3 angles of the triangle whose arms on extension formed the CSC triangle. After the generation of CSC triangle, and the Gonial angles for both the sides; the data was entered in excel sheet for statistical analysis.

The data was analyzed using SPSS for Windows [ver 26.0, IBM Corp., Armonk, NY]. The equation to predict the age according to independent variables was derived using the linear regression and discriminant analysis was used to derive an equation that classifies the values into either sex. Results were presented using graphs and tables. In addition, continuous data was compared using unpaired and paired *t*-test.

## 3 Results

On comparing the statistical data, it was found that the mean age of male participants was more than that of female participants, which was statistically significant (p = 0.03) (Table 1). Condylar, Coronoidal and Sigmoidal angles of both sides and Gonial angle of the right side was comparable between males and females (p > 0.05) (Table 2).

**Table 1:** Comparison of mean age of study participants.

	Number	Mean	SD	t	P value
Males	100	38.1	17.06	2.3	P = 0.03∗
Females	100	33.2	12.2		

SD - standard deviation; ∗statistically significant using unpaired *t*-test.

**Table 2:** Comparison of mean values of Condylar, coronoid, Sigmoidal, and Gonial angle between sexes.

		Males		Females		P value
		Mean	SD	Mean	SD	
Condylar Angle	Right	34.05	5.3	32.8	5.7	P = 0.13
	Left	35.2	5.5	35.6	5.6	P = 0.6
Coronoidal Angle	Right	47.3	8.5	48.7	10	P = 0.29
	Left	46.08	7.4	47.2	7.6	P = 0.29
Sigmoidal Angle	Right	96.6	11.7	96.8	12.3	P = 0.93
	Left	96.5	10.3	95.7	9.6	P = 0.58
Gonial Angle	Right	129.7	8.4	128.9	6.3	P = 0.44
	Left	127.08	8.1	129.3	7.07	P = 0.03∗

SD - standard deviation; ∗statistically significant using the unpaired *t*-test.

On comparing the Coronoidal, Condylar and Sigmoidal angles with respect to right and left sides, it was found that the Condylar angle had a higher score on the left side compared to the right side (p = 0.01) with the Coronoidal angle having a higher score on the right side compared to the left side (p = 0.04). The Sigmoidal and the Gonial angle were comparable between the right and left sides (p > 0.05) (Table 3).

**Table 3:** Comparison of Mean Condylar, Coronoidal, Sigmoidal, and Gonial angles between right and left sides.

			Mean	SD	t	P value
Condylar Angle	Right	200	33.4	5.5	−4.5	P = 0.01∗
	Left	200	35.44	5.5		
Coronoidal Angle	Right	200	48.05	9.3	2.03	P = 0.04∗
	Left	200	46.6	7.5		
Sigmoidal Angle	Right	200	96.7	12.06	0.7	P = 0.46
	Left	200	96.1	9.9		
Gonial Angle	Right	200	129.3	7.4	1.8	P = 0.07
	Left	200	128.2	7.7		

SD - standard deviation; ∗statistically significant using the paired *t*-test.

A P value of 0.47 with the variables was obtained through the regression analysis 0.1.8 % of the variance was explained by the model (Table 4). The least standard error of 0.17 was observed in the Gonial angle and the variable with the highest standard error was the Condylar angle 0.376 (Table 5).

**Table 4:** Coefficient of dependent variables between residuals and regression with model summary.

	Sum of Squares	df	Mean Square	F	P value	R	R^2^	SEE
Regression	794	4	198.5	0.877	P = 0.47			
Residuals	44149.1	195	226.4		NS	0.133	0.018	15.04
Total	44943.18	199

**Table 5:** Regression coefficient for variables used in the model.

	B	SE	t	P value
Constant	38.53	53.2	0.7	P = 0.4
Condylar Angle	0.107	0.37	0.28	P = 0.7
Coronoidal Angle	0.182	0.27	0.67	P = 0.5
Sigmoidal Angle	0.189	0.25	0.74	P = 0.45
Gonial Angle	−0.26	0.17	−1.5	P = 0.12

The equation for age estimation:

**Age (overall)** = 38.53 + 0.107 X Condylar angle +0.183 X Coronoidal angle + 0.189 X Sigmoid angle + 0.189 X Gonial angle

Standard error of the estimate (SEE) = The difference between actual age and predicted age by the equation is 15.04 years.

A P value of 0.7 and 0.1 was obtained through regression analysis for males and females respectively. 1.8 % and 7.8 % of the variance was explained by the model for males and females (Table 6, Table 7).

**Table 6:** Coefficient of dependent variables between residuals and regression with model summary for males and females.

		Sum of Squares	df	Mean Square	F	P value	R	R^2^	SEE
Males	Regression	508.9	4	127.2	0.427	P = 0.7			
	Residuals	28318.4	95	298.08		NS	0.133	0.018	17.2
	Total	28827.3	99						
Females	Regression	1160.2	4	290.06	2	P = 0.1			
	Residuals	13725.4	95	144.4		NS	0.27	0.078	12.02
	Total	14885.7	99

**Table 7:** Regression coefficient for variables used in the model.

Sex		B	SE	t	P value
Males	Constant	43.2	73.4	0.59	P = 0.557
	Condylar Angle	0.046	0.6	0.075	P = 0.94
	Coronoidal Angle	0.28	0.35	0.76	P = 0.44
	Sigmoidal Angle	0.015	0.35	0.042	P = 0.96
	Gonial Angle	−0.167	0.24	0.042	P = 0.48
Females	Constant	−38.3	85.2	−0.45	P = 0.65
	Condylar Angle	0.45	0.48	0.9	P = 0.35
	Coronoidal Angle	0.59	0.43	1.3	P = 0.17
	Sigmoidal Angle	0.75	0.41	1.8	P = 0.07
	Gonial Angle	−0.34	0.23	−1.45	P = 0.14

Gonial angle had the least standard error for males and females, while Condylar angles had the highest standard error.

**Age (Males)** = 43.2 + 0.046 X Condylar angle + 0.286 X Coronoidal angle + 0.015 X Sigmoidal angle – 0.16 X Gonial angle

Standard error of the estimate (SEE) = The difference between actual age and predicted age by the equation is 17.2 years.

**Age (Females)** = −38.3 + 0.45 X Condylar angle + 0.56 X Coronoidal angle + 0.75 X Sigmoidal angle – 0.34 X Gonial angle

Standard error of the estimate (SEE) = The difference between actual age and predicted age by the equation is 12.02 years.

The eigenvalue of 0.02 was obtained in the present analysis by classifying values based on sex (Table 8). Thus, the canonical functional discriminant equation is:

**Sex** = 27.133–0.008 X Condylar angle +0.168 X Coronoidal Angle +0.07 X Sigmoidal Angle +0.099 X Gonial Angle

**Table 8:** Summary of Discriminant functions.

Eigen Value	Canonical correlation	Wilk's Lamba	P value
0.02	0.14	0.98	P = 0.423

The discriminant functions at group centroids (group means) were −0.141 for males and 0.141 for females; Compared to the condylar (−0.008) and Sigmoidal angles (0.07), the Coronoidal angle (0.168) and Gonial angle (0.099) demonstrated the largest positive coefficients among the variables, suggesting a comparatively higher contribution to sex discrimination. A small and inverse contribution to the discriminant function was suggested by the Condylar angle's negative coefficient. The discriminant equation's intercept was represented by the constant (−27.133) (Table 9). A discriminant score less than 0 classifies the values as males, and a discriminant score more than 0 classifies the values as females. In addition, it was found that the accuracy of discrimination of data according to sex was 52 % for males and 56 % for females (Table 10).

**Table 9:** Discriminant analysis for the prediction of sex.

Functional Coefficients	
Condylar Angle	−0.008
Coronoidal Angle	0.168
Sigmoid Angle	0.07
Gonial Angle	0.099
Constant	−27.133

**Table 10:** Classification accuracy of groups.

Actual Group	Number of cases	Predicted	
		Males	Females
Males	100	48	52
Females	100	44	56

## 4 Discussion

Identification of the sex of an unidentified skeleton can be challenging, particularly in circumstances of explosions, conflict, or mass calamities. Identification of skeletal remains is crucial in forensic medicine and anthropology, particularly in criminal investigations. Understanding the changes in the mandible based on age, sex, and race can assist surgeons, physicians, and anthropologists in accurately interpreting diagnostic results in living individuals.^9^ Under these circumstances, establishing the role of mandibular morphology is essential when other skeletal components are absent or fragmented. Most frequently, the pelvis and skull are examined skeletally to determine sex, with the mandible being utilised to examine sexual dimorphism in fractured bones. When these bones are impaired or absent, the mandible serves as a valuable alternative due to its robustness and preservation potential. Human identification from a mandible is crucial in anthropological research and medical-legal cases since it is the biggest, strongest, and most mobile component of the skull.^10^ Therefore, its fundamental anatomy often provides reliable indicator for sex determination and age estimation in both anthropological and forensic contexts. The mandibular condyle and ramus exhibit the largest sexual dimorphism, with significant variations in size and remodeling during development.^11^

The mandibular condyle joins the ramus and body. The condyle measures 20 mm in length medio-laterally and 8–10 mm thick antero-posteriorly on an average. Size and shape variations can be natural or pathological in nature. The mandible has numerous variations, including flattened, rounded, or convex, superior aspect flat, medio-lateral aspect convex. Such variations usually result from genetic, functional, or environmental factors influencing the mandibular growth. The sigmoid notch is the increased space between the condyle and the mandibular body, while the coronoid process, which means "crow," is defined as one of the bony processes of the mandibular ramus. The morphometric correlations of these anatomical features—in particular, the condyle, coronoid process, and sigmoid notch are regularly examined. Several studies have analyzed the morphological aspects of these processes with very few literature evidence of the assessment of the angles formed by these processes which project an anatomic interrelationship between these structures which may reflect the advancing age changes and possibly difference between the sexes in the arena of Forensic Odontology.^12^ This highlights a gap in current research and the need for further exploration of angular measurements as indicators of age changes and in lieu of sexual dimorphism in diverse groups of specific populations.

As a suitable screening method for identifying dental disorders, OPGs are frequently used and advised by medical professionals. The ability to quantify several landmarks from skeletal remains makes it an adjuvant radiography tool for sex identification. Among the many benefits of panoramic photographs are their rapid collection, low radiation dosage to patients, and wide coverage. The absence of interference from overlay images is another benefit. Additionally, image enlargement, contrast, and brightness enhancement provide a precise and trustworthy method for measuring specific areas.^13^ Thus, panoramic radiographs not only aid in clinical diagnosis but also serve as valuable adjuncts in Forensic Odontology as well as Anthropology.

Metric research studies on radiographs are often shown to be preferable in terms of reliability, correctness, and objectivity when it comes to skeleton sex determination. Ante mortem radiographs serve as the basis for identifying human remains in forensic anthropology with utilisation of a calibrated measurement tool. Comparison between ante mortem and post mortem images provides a scientific basis for identification in disaster victim investigations. The prevalence of panoramic radiography offers a great opportunity to study sexual dimorphism and estimate the age of individuals in particular populations. Additionally, these radiographic approaches can be correlated with other identification methods for comprehensive analysis. Soft tissues, dental records, tooth DNA analysis, and the morphology and metric features of the skull and mandible can all be used to determine the sex of an unidentified person.^14^

In our study, the mean age of participants was 38.1 ± 17.06 for males and 33.2 ± 12.2 for females. The mean Condylar (34.05±5.3^0^ and 35.2±5.5^0^), Coronoidal (47.3 ± 8.5^0^and 46.08±7.4^0^) and Sigmoidal (96.6±11.7^0^ and 96.5±10.30^0^) angles and Gonial angle of the right side (129.7±8.4^0^) were comparable among males and females which was in accordance with the study conducted by Ingaleshwar P et al.^15^

On comparing the sides, the Condylar angle on the left side (35.44±5.5^0^) was higher than the right side (33.4±5.5^0^) with the Coronoidal angle on the right side (48.05±9.3^0^) being higher than left side (46.6±7.5^0^) which was found to be statistically significant. The asymmetry between the sides indicates the possibility of functional domination, or developmental variability influencing mandibular morphology. This was in line with the findings attained from the study conducted by Farahani et al.^16^ who showcased the Condylar-Coronoidal angle to be comparable bilaterally. The Gonial angle was comparable in our study between right (129.3 + 7.4 ^0^) and left side (128.2 + 7.7 ^0^) which was in accordance with the study by Abuhijleh et al.^17^

Although a novel concept, the angles of CSC triangle can be a useful tool for determining age and sex independent of dental parameters, especially in forensic utility. The changes could be attributed to the sample size variation as well as geographical discrepancies noted amongst the population. In the current investigation, the measurements obtained showed statistically significant difference between males and females. The mandibular development stages, developmental status, growth rates, and duration may all be responsible for this discrepancy. Mandibular morphology can be influenced by a number of factors, including dietary patterns, hormone fluctuations, occlusal forces, and masticatory forces. Of these, hormonal influences—specifically, sex hormones that impact bone metabolism may be a significant factor, though more research is needed in this area.^18^

In our study the Sigmoidal angle was evaluated which was not studied earlier for forensic purposes. In order to broaden the application of morphometric analysis, the Sigmoidal angle was incorporated in the formulation of the CSC triangle, aiming to assess the potential influence exerted by these angular parameters in age estimation and sex determination if any.

The influence of masticatory muscles and sex-specific hormonal variations that impact bone metabolism could provide the possible explanation for the variation of Gonial angles noticed between males and females as age advances. Moreover, the relationship between functional biomechanics and bone remodeling could further add clarity to these findings. The variations in angle measurement observed from this investigation corroborated the inherent adaptability of the human mandible with age and sex of individuals.^8^ Therefore, the findings highlight the intricacy of deviation in age related anatomy of developing mandible and the necessity of morphometric databases tailored to specific populations in order to ensure near precise forensic identification.

## 5 Limitations of the study

This study explored an unnavigated arena of Condylar, Sigmoidal and Coronoidal angle; the formed CSC Triangle along with Gonial angle for age and sex assessment. The small sample size and narrow representation of racial and ethnic groupings among the chosen subjects are the study's limitations, which has caused Low predictive accuracy and Population specificity. The use of 2D imaging for 3D structures is another limitation that warrants a similar study with 3D imaging modality. Further research, using the above study parameters to devise a formula, may be performed on a larger sample size of specific population groups to produce a database for forensic utility to strengthen the claim.

## 6 Conclusion

The study found a substantial difference in Gonial, Condylar, Sigmoidal and Coronoidal angles with the utilisation of Panoramic Radiography by engineering the CSC triangle. The variations exhibited by the angular components of this triangle between males and females as well as with respect to the borders of the triangle, could be utilised in the field of Forensic Odontology with the utilisation of radiographic imaging modalities covering into three dimensional radiographs. The equation obtained through this study could be utilised for a larger population henceforth enabling the determination of the age with the help of these angular parameters, especially when teeth are missing, paving the way for a varied onset for age estimation methods.

## Patient's/Guardian's consent

As the research was retrospective, Patient's/Guardian's Consent was not required for the study.

## Declaration of competing interest

The authors declare that they have no known competing financial interests or personal relationships that could have appeared to influence the work reported in this paper.
